# Comparative Proteomic Analysis of the Response of Maize (*Zea mays* L.) Leaves to Long Photoperiod Condition

**DOI:** 10.3389/fpls.2016.00752

**Published:** 2016-06-02

**Authors:** Liuji Wu, Lei Tian, Shunxi Wang, Jun Zhang, Ping Liu, Zhiqiang Tian, Huimin Zhang, Haiping Liu, Yanhui Chen

**Affiliations:** ^1^Henan Agricultural University and Synergetic Innovation Center of Henan Grain CropsZhengzhou, China; ^2^Key Laboratory of Physiological Ecology and Genetic Improvement of Food Crops in Henan ProvinceZhengzhou, China; ^3^Food Crops Research Institute, Henan Academy of Agricultural ScienceZhengzhou, China; ^4^Department of Biological Science, Michigan Technological UniversityMichigan, MI, USA

**Keywords:** proteomic analysis, maize leaves, introgression line, iTRAQ, long photoperiod, cytoscape, circadian

## Abstract

Maize (*Zea mays L.*), an important industrial material and food source, shows an astonishing environmental adaptation. A remarkable feature of its post-domestication adaptation from tropical to temperate environments is adaptation to a long photoperiod (LP). Many photoperiod-related genes have been identified in previous transcriptomics analysis, but proteomics shows less evidence for this mechanism of photoperiod response. In this study, we sampled newly expanded leaves of maize at the three- and six-leaf stages from an LP-sensitive introgression line H496, the donor CML288, LP-insensitive inbred line, and recurrent parent Huangzao4 (HZ4) grown under long days (15 h light and 9 h dark). To characterize the proteomic changes in response to LP, the iTRAQ-labeling method was used to determine the proteome profiles of plants exposed to LP. A total of 943 proteins differentially expressed at the three- and six-leaf stages in HZ4 and H496 were identified. Functional analysis was performed by which the proteins were classified into stress defense, signal transduction, carbohydrate metabolism, protein metabolism, energy production, and transport functional groups using the WEGO online tool. The enriched gene ontology categories among the identified proteins were identified statistically with the Cytoscape plugin ClueGO + Cluepedia. Twenty Gene Ontology terms showed the highest significance, including those associated with protein processing in the endoplasmic reticulum, splicesome, ribosome, glyoxylate, dicarboxylate metabolism, L-malate dehydrogenase activity, and RNA transport. In addition, for subcellular location, all proteins showed significant enrichment of the mitochondrial outer membrane. The sugars producted by photosynthesis in plants are also a pivotal metabolic output in the circadian regulation. The results permit the prediction of several crucial proteins to photoperiod response and provide a foundation for further study of the influence of LP treatments on the circadian response in short-day plants.

## Introduction

Maize (*Zea mays L.*) is a key food source and industrial material that has rapidly spread in cultivation since originating in Southern Mexico 6000–10,000 years ago from Balsas teosinte (*Zea mays* ssp. parviglumis; Matsuoka et al., 2002). Balsas teosinte required short-day (SD) conditions for flowering (Emerson, 1924). One remarkable determinant enabling the spread of maize across latitudes was the post-domestication adaptation to changing in daylight hours (Piperno et al., 2009; van Heerwaarden et al., 2011). Under the longer days experienced at higher latitudes, tropical maize cultivars do not flower or show delayed flowering (Betran et al., 2003). Plants integrate signals from endogenous regulatory pathways or the environment to modulate the timing of flowering (Colasanti and Coneva, 2009). In the model plant *Arabidopsis thaliana*, numerous components associated with the plant circadian clock and photoperiod have been studied to understand the regulation and molecular mechanism of flowering in higher plant (Matsubara et al., 2008; Kumimoto et al., 2010; Lazakis et al., 2011; Knuesting et al., 2015). However, only a small number of genes such as ZCN1, ZCN8, and conzl identified by the *Arabidopsis* orthologues AtTFL1, AtFT, and AtCO, respectively (Danilevskaya et al., 2008; Matsubara et al., 2008; Miller et al., 2008; Lazakis et al., 2011), have been shown to be involved in the regulation of flowering time and the vegetative to reproductive transition in maize. Recently, numerous quantitative trait loci (QTLs) were affecting flowering date and response to photoperiod were detected, each with a small effect (Buckler et al., 2009). The photoperiod response may be influenced by only a small number of these flowering-time QTLs, including ZmCCT which encodes a CCT domain-containing protein (Ducrocq et al., 2009; Coles et al., 2010; Hung et al., 2012). Previously, our research group identified CACTA-like transposable elements in ZmCCT, which were shown to attenuate the photoperiod sensitivity and to accelerate the post-domestication spread of maize (Yang L. T. et al., 2013).

The circadian system influences expression of a substantial fraction of the genes in a variety of species because of the diversity of clock outputs. Approximately 10 and 30% of genes are estimated to be regulated by the circadian system in mammals and plant, respectively (Panda et al., 2002; Michael and McClung, 2003; Covington et al., 2008). Circadian rhythms are entrained by environmental signals, such as temperature and light, and by endogenous sugar production by photosynthesis to enable a plant to adapt the local environment (Harmer et al., 2000; Haydon et al., 2013).

Recent studies on photoperiod response have highlighted the emergence of proteomic analysis as a promising tool. To our knowledge, our group is responsible for the only previous proteomic analysis of photoperiod responses in maize, using classical 2-D electrophoresis (2-DE) combined with mass spectrometry (MS; Wang et al., 2015). In that study, however, only a few proteins responsive to long photoperiod (LP) were identified (Wang et al., 2015). And in our study, we also used the bioinformatics tools WEGO (GO annotation) and Cytoscape (v3.0.2) plugin ClueGO + Cluepedia v2.1 (GO-KEGG network) for functional classification and enrichment analysis, and argue that photoperiod response to LP will show a close relationship with protein synthesis, metabolism process, post-transcriptional regulation and mitochondrial outer membrane. None of this is included in the Wang et al. (2015) article. For each functional category, we identified more proteins compared with the Wang et al. (2015) study. Especially for these “circadian” related proteins. Therefore, the current study lays a foundation for future elucidation of the protein network regulatory mechanism underlying the photoperiod response.

*Stevia rebaudiana* plants grown under long-photoperiod (LP) conditions show increased leaf size, internode length and dry weight, but reduced intervals between successive leaf pairs, compared with plants grown under SD (Metivier, 1979). However, few studies on proteomic fluctuations in response to LP in the maize leaf have been undertaken. To clarify the mechanism involved in alterations of the proteome, in the present study we collected newly expanded third and sixth leaves from the photoperiod-insensitive maize inbred line Huangzao4 (HZ4) and the photoperiod -sensitive inbred line H496 obtained through crossing the recurrent parent of HZ4 with CML288 (non-recurrent parent). A total of 5259 proteins and 14 proteins directly related to the photoperiod were identified by isobaric tags for relative and absolute quantitation (iTRAQ) labeling in response to the LP condition.

## Materials and methods

### Plant materials

The near-isogenic lines H496, which is highly photoperiod-sensitive, was derived from a cross between HZ4 (the recurrent parent) and a tropical maize inbred line, CML288, The latter was acquired from the National Maize and Wheat Improvement Center in Mexico, whereas HZ4 is a representative of the Chinese Tangsipingtou heterotic group. Four plants were grown in each 15 cm pots under LP conditions (15/9 h, light/dark; Ku et al., 2011). Newly developed third and sixth leaves were collected for proteomic analysis. All leaf samples were immediately frozen in liquid nitrogen stored at −80°C until use.

### Sucrose and glucose measurement

Fresh leaf material of HZ4 and H496 sampled at three- and six-leaf stages (15/9 h, light/dark) were separately ground to fine powder with a mortar and pestle in liquid nitrogen. The sucrose and glucose content was determined by enzyme-coupled reactions using the Sucrose/D-Glucose/D-Fructose assay kit (R-Biopharm, Darmstadt, Germany) as described by Thalor et al. (2012). A sample (200 mg) of the powder was immediately boiled with 600 μl distilled water for 15 min in a water bath. After centrifugation (16,000 × g, 15 min at 4°C), 100 μl of the supernatant was used for absorbance determination in the sucrose assay by the spectrophotometer (Hitachi U-2900, Hitachi, Tokyo, Japan).

### Fe content measurement

Leaves at the three- and six-leaf stages of the two maize inbreds were collected in three biological replications for Fe concentration analyses. One hundred microgram of the leaves were dried for 2–3 days at 70°C, then digested with 1 ml of 13 M HNO3 and 1 ml of 8.8 M H2O2 (Wako, Japan) at 220°C for 20 min using MARS Xpress oven (CEM, USA) as described by Masuda et al. (2008); After digestion, the samples were diluted to 5 ml and analyzed using a SPS1200VR ICPAES (Seiko, Japan).

### Protein digestion and iTRAQ labeling

Digestion of protein was carried out in accordance with the filter-aided sample preparation (FASP) protocol used by Wisniewski et al. (2009). Briefly, the method used was as follows. For each sample, 200 μg proteins were suspended in 30 μl STD buffer (4% SDS, 150 mM Tris-HCl, 100 mM DTT, pH 8.0), incubated in boiling water for 5 min and then cooled to room temperature. The DTT (detergent) and other low-molecular-weight components were diluted with 200 μl UA buffer (150 mM Tris-HCl, 8 M urea, pH 8.0) and transferred by repeated ultrafiltration (Microcon units, 30 kD). Next, 100 μl of 0.05 mol·L-1 iodoacetamide (IAA) was added to the UA buffer to block the reduced cysteine residues. The mixture was incubated in darkness for 20 min. The filters were washed three times with 100 μl UA buffer and then twice with 100 μl DS buffer (50 mM triethylammonium bicarbonate, pH 8.5). Finally, 2 μg trypsin (Promega, Madison, USA) was used to digest the protein suspensions in 40 μl DS buffer at 37°C overnight. The digested peptides were collected as a filtrate. The concentration of peptides was measured by UV light spectral density at 280 nm using an extinction coefficient of 1.1 of 0.1% (g/l) solution, which was calculated based on the frequency of tyrosine and tryptophan in vertebrate proteins.

For labeling, the digested products of the peptide mixture were labeled with 8plex iTRAQ® Reagents following the manufacturer's instructions (Applied Biosystems). Briefly, 70 μl of ethanol was used to dissolve each iTRAQ reagent and then the solution was combined with respective peptide mixture. The samples were labeled (496-6Y)-113, (496-3Y)-114, (HZ4-6Y)-115, and (HZ4-3Y)-116 and were multiplexed and vacuum-dried.

### Peptide fractionation with strong cation exchange (SCX) chromatography

An AKTA purifier system (GE Healthcare) was used to fractionate iTRAQ-labeled peptides. Reconstitution and acidification of the dried peptide mixture were performed using 2 ml of buffer A [10 mM KH2PO4 in 25% (v/v) acetonitrile, pH 2.7]. The products were loaded onto a polysulfethyl 4.6 × 100 mm column (5 μm, 200 Å; PolyLC Inc., Columbia, MD, USA). A gradient of 0–10% buffer B [10 mM KH2PO4 in 25% (v/v) acetonitrile, 500 mM KCl, pH 2.7] was used for elution of the peptides at a flow rate of 1 ml min-1 for 2 min, 10–20% buffer B for 25 min, 20–45% buffer B for 5 min, and 50–100% buffer B for 5 min. The elution was monitored by absorbance at 214 nm, and fractions were collected at 1-min intervals. All collected fractions (~30) were finally grouped into 10 pools and desalted on C18 cartridges [Empore™ SPE cartridges C18 (standard density), bed i.d. 7 mm, volume 3 ml, Sigma]. After concentration by vacuum centrifugation, each fraction was reconstituted in 40 μl of 0.1% (v/v) trifluoroacetic acid. Before liquid chromatography-tandem mass spectrometry (LC-MS/MS) analysis, all samples should be stored at −80°C.

### LC-ESI MS/MS analysis

Q Exactive™ mass spectrometer coupled with an Easy-nLC chromatography system (Proxeon Biosystems, now Thermo Fisher Scientific) were used to perform the following experiments. For nano LC-MS/MS analysis, totally 10 μl of each fraction was used. The peptide mixture (5 μg) was loaded into the C18 reversed-phase column (15 cm length, 75 μm id) packed in-house with RP-C18 5 μm resin in buffer A (0.1% formic acid) and separated by buffer B with a linear gradient (0.1% formic acid and 80% acetonitrile) at the flow rate of 250 nl/min controlled by an Intelli Flow Technology controller over 140 min. Data-dependent top 10 method, which dynamically chose the most abundant precursor ions from the survey scan (300–1800 m/z) for HCD fragmentation, was used to acquire the MS data. Predictive automatic gain control (pAGC) was applied to determinate the target value. Dynamic exclusion duration was 60 s. Resolution for survey scans was set to 70,000 at m/z 200, while for HCD spectra, the resolution 17,500 at m/z 200. 30 eV was applied for normalized collision energy and 0.1% was defined for the underfill ratio which specifies the minimum percentage of the target value likely to be reached at maximum fill time. Peptide recognition mode was enabled during the running of the instrument.

### Sequence database search and data analysis

MASCOT engine (Matrix Science, London, UK; version 2.2) was embedded into the Proteome Discoverer 1.3 (Thermo Electron, San Jose, CA, USA) for searching MS/MS spectra against the decoy database and UniProt Plant database (134,648 sequences, downloaded on May 5, 2013). The following parameters were used for identifying proteins. ±20 ppm is set for peptide mass tolerance, 0.1 Da for MS/MS tolerance, 2 for missed cleavage, enzyme is trypsin, fixed modification: carbamidomethyl (C), iTRAQ4/4plex(K), iTRAQ4/4plex(N-term), Variable modification: oxidation (M), iTRAQ4plex (Y), 20 ppm for integration window tolerance, 0 for minimum quan value threshold, 2 for fold change threshold for up/down regulation, 100 for maximum allowed fold change, and FDR is no more than 0.05 (Sandberg et al., 2012). For iTRAQ studies, we used confidence scores >1.2-fold, FDR ≥0.05, as the qualification criterion, which corresponded to a peptide confidence level of 95% (Yang Q. et al., 2013).

### Bioinformatics

As described by Ye et al. (2006), Gene Ontology (http://www.geneontology.org/) and WEGO (http://wego.genomics.org.cn/) online tools were used for functional analysis of the proteins. The statistically enriched gene ontology (GO) categories for the identified protein interactome were determined by Cytoscape (v3.0.2) plugin ClueGO + Cluepedia v2.1.3 (Bindea et al., 2009, 2013). The analysis was carried out using the proteins identified in the three-leaf and six-leaf stage in these two inbred lines. Biological processes, subcellular locations, molecular function and KEGG (Kyoto Encyclopedia of Genes and Genomes) pathways (Kanehisa and Goto, 2000), which were inferred electronic annotation and experimental data, were all in the identified GO categories. A minimum level of 5 and a maximum level of 11 were set as the GO level interval with a minimum of two genes per category. And a right-sided hypergeometric test for enrichment analysis was elicited applying against the ClueGO *Z. Mays* reference genome.

### *post-hoc* test

To verify the main variable contributing to the differences, a two-way analysis of variance with *post-hoc* test was performed using SAS software (SAS Institute Inc., Cary, NC, USA).

## Results and discussion

### Phenotypes and growth parameters between HZ4 and H496 in maize

**Figure 1:**
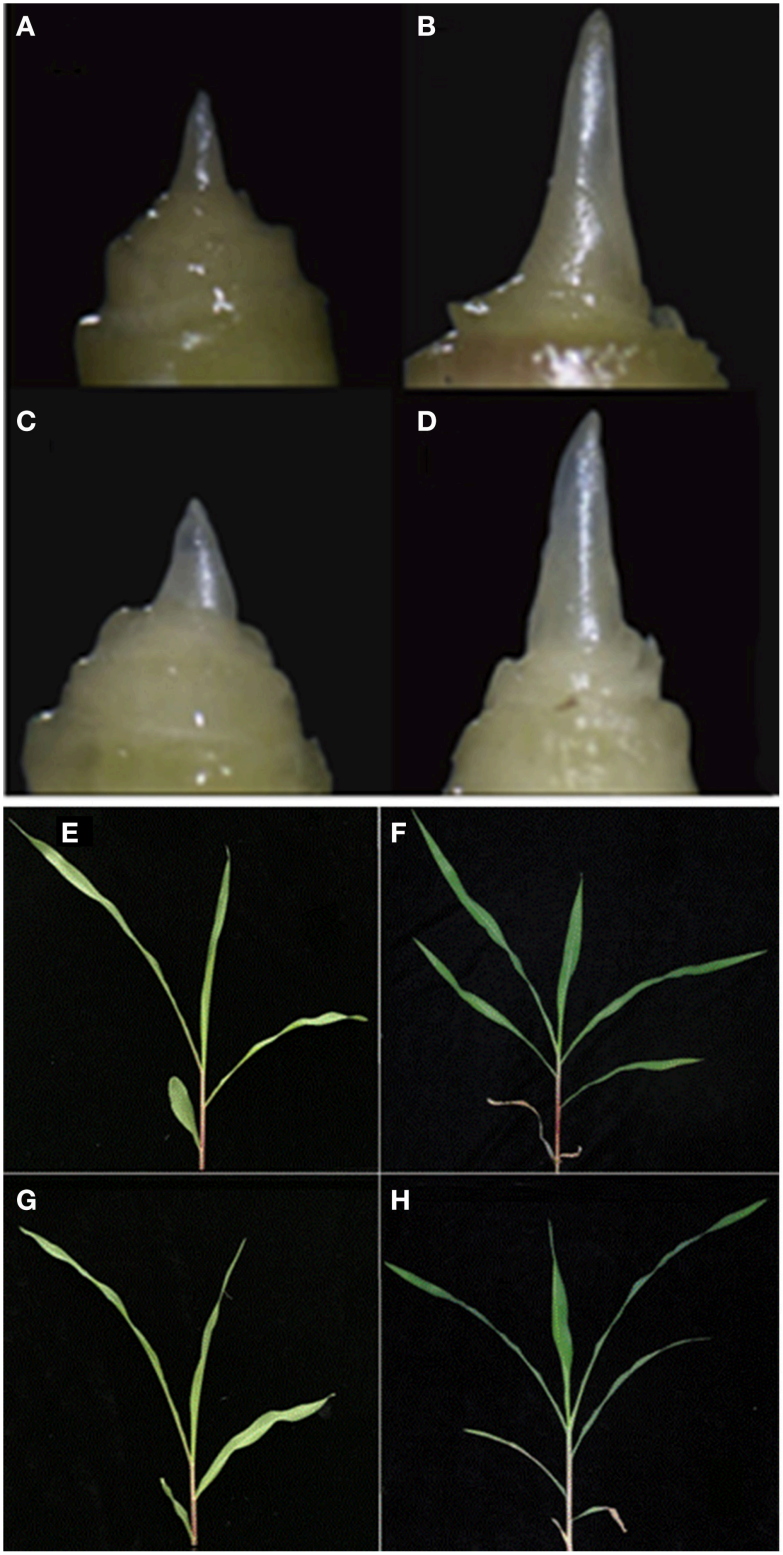
**Morphology of the shoot apex and leaves at the three- and six-leaf stages of maize inbred line HZ4 and H496 grown under long-photoperiod conditions. (A–D)** Shoot apex of HZ4 at the three-leaf stage **(A)** and six-leaf stage **(B)**. Shoot apex of H496 at the three-leaf stage **(C)** and six-leaf stage **(D). (E–H)** Leaves of HZ4 at the three-leaf stage **(E)**, and six-leaf stage **(F)**; shoot apex of H496 at three-leaf stage **(G)**, and six-leaf stage **(H)**.

To examine the protein changes response to the photoperiod in maize leaves, plant phenotypes were periodically observed in the two inbred lines HZ4 and H496 under LP at the three- and six-leaf stages, and the individual samples were collected. Under the LP condition, plants of the H496 line were considerably taller than those of the HZ4 line. HZ4 plants showed less photoperiod sensitivity than H496 plants, in which flowering was delayed by 1 week (Table 1). The two lines showed similar leaf and shoot apex phenotypes at each of the three- and six-leaf stages (Figure 1).

**Table 1:** **Phenotypes of the maize inbred line HZ4 and near-isogenic line H496 grown under long-photoperiod conditions**.

**Name**	**Plant Height**	**Tasseling stage**	**Silking**
HZ4	127	60(0.30)	70(0.32)
H496	164	67(0.48)	79(0.26)

Previously, the number of leaves and morphologies of the shoot apical meristem were used to indicate the inductive phase changes of photoperiod sensitivity in maize. This showed that the juvenile vegetative stage was completed between the four- and five-leaf stages in HZ4 and CML288 under LP condition (Wu et al., 2008). In the present study, we observed that the shoot apical meristem was elongated in the six-leaf stage in both HZ4 and H496 inbred lines (Figures 1E–H), which proved that plants were in different developmental phases at the three- and six-leaf stages. Thus, it seems that examination of photoperiod-sensitive phenotypic traits is significant during improvement of maize germplasm.

### iTRAQ analysis of protein expression in response to LP condition

**Figure 2:**
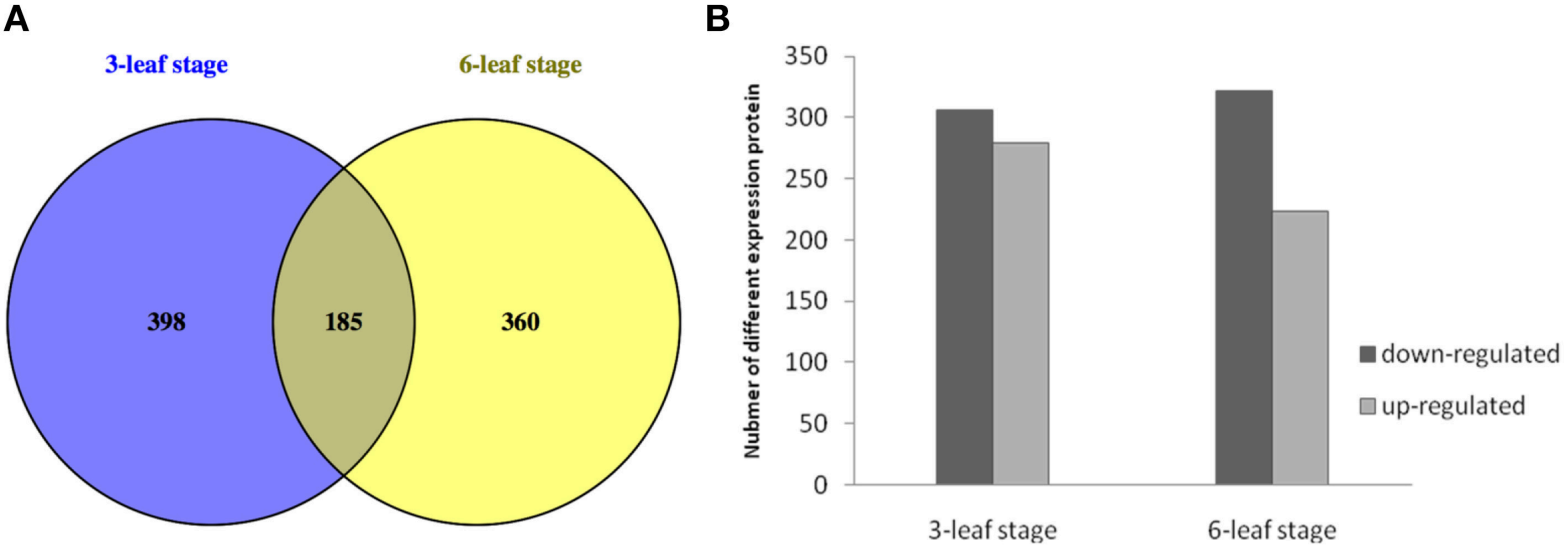
**Expression patterns between HZ4 and H496 at the three- and six-leaf stage grown under long-photoperiod conditions. (A)** Venn diagram of differentially expressed proteins identified in HZ4 and H496 at the three- and six-leaf stages. **(B)** Number of differentially expressed proteins identified in HZ4 and H496 at three- and six-leaf stages.

In this study, 23,767 unique peptides were analyzed. A total of 5259 proteins were identified by MS/MS (Table S 1). The peptides of the identified proteins are listed in Tables S 1, S 2. According to the criteria for recognition of differentially expressed proteins (fold change ratio >1.2 and *p* < 0.05), 943 proteins differentially expressed between H496 and HZ4 were identified, of which 185 proteins were differentially expressed at both developmental stages (Table S 3), 398 proteins showed differential expression at the three-leaf stage (Table S 4), and 360 proteins were only differentially expressed at the six-leaf stage (Figure 2A, Table S 5). The results showed that the difference between the inbred lines lead to the changes of the proteins. Of the differentially expressed proteins, 278 upregulated and 305 downregulated proteins were identified at the three-leaf stage (Figure 2B, Tables S 3, S4), and 223 upregulated and 322 downregulated proteins were detected at the six-leaf stage (Figure 2B, Tables S 3, S5).

Analysis of variance confirmed that there were significant differences in protein expression between the inbred lines, but there was no significant difference between the two developmental stages (Table S 6). Previously, our group used the H496-10 line which was produced after one less generation of back-crossing with HZ4 than H496 via gel-based proteomic approach to provide novel insights into the influences of long-photoperiod treatments on short-day plants (Wang et al., 2015), but there are significant differences and considerable novelty in this study, we choosed three- and six-leaf stage as two distinct phases in order to verify what proteins change in expression before and after the onset of the induction phase according to Wu et al. (2008). And 943 proteins differentially expressed were identified, while only 22 differentially expressed proteins between HZ4 and H496-10 (Wang et al., 2015). Clearly, this current report by iTRAQ method identified many additional proteins and presents further evidence with which to understand the photoperiod response in maize.

### Functional characterization of protein interaction network

**Figure 3:**
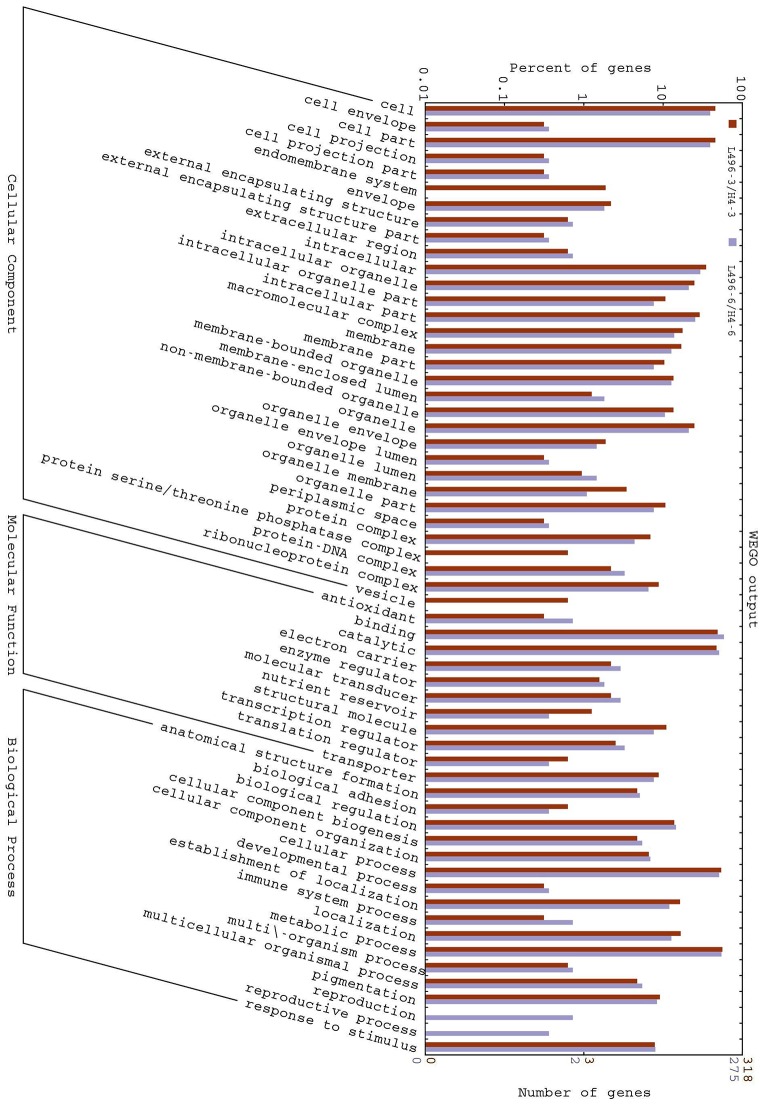
**Gene Ontology (GO) classification of differentially expressed proteins at the three- and six-leaf stages identified by iTRAQ-labeling experiments between HZ4 and H496**. The differentially expressed proteins are grouped into three hierarchically structured GO terms: biological process, cellular component, and molecular function. The *y*-axis indicates the number and percent of proteins in each GO term.

We analyzed the GO annotation of the 943 proteins that were expressed differentially at the three- and six-leaf stages of H496 compared with HZ4 (Tables S 3–S 5) to gain insights into the functions of the proteins and the mechanism involved in the photoperiod pathway. The WEGO tool was used to plot the distribution of GO annotations (Figure 3). The differentially expressed proteins were grouped into three hierarchically structured GO terms, namely biological process, cellular component, and molecular function. The differentially identified proteins were subcategorized into 16 main hierarchically structured GO classifications including 4 biological processes, 10 cellular components, and 2 molecular functions (Figure 3). Specifically, “metabolic process” and “cellular process” were highly represented in “biological process”; “cell part”, “cell” and “intracellular” were incorporated in “cellular component”; and “binding,” “catalytic activity” were represented in “molecular function” (Figure 3). This analysis indicated that the identified proteins involved in these GO categories may play the most important roles in regulation of the photoperiod response to LP.

**Figure 4:**
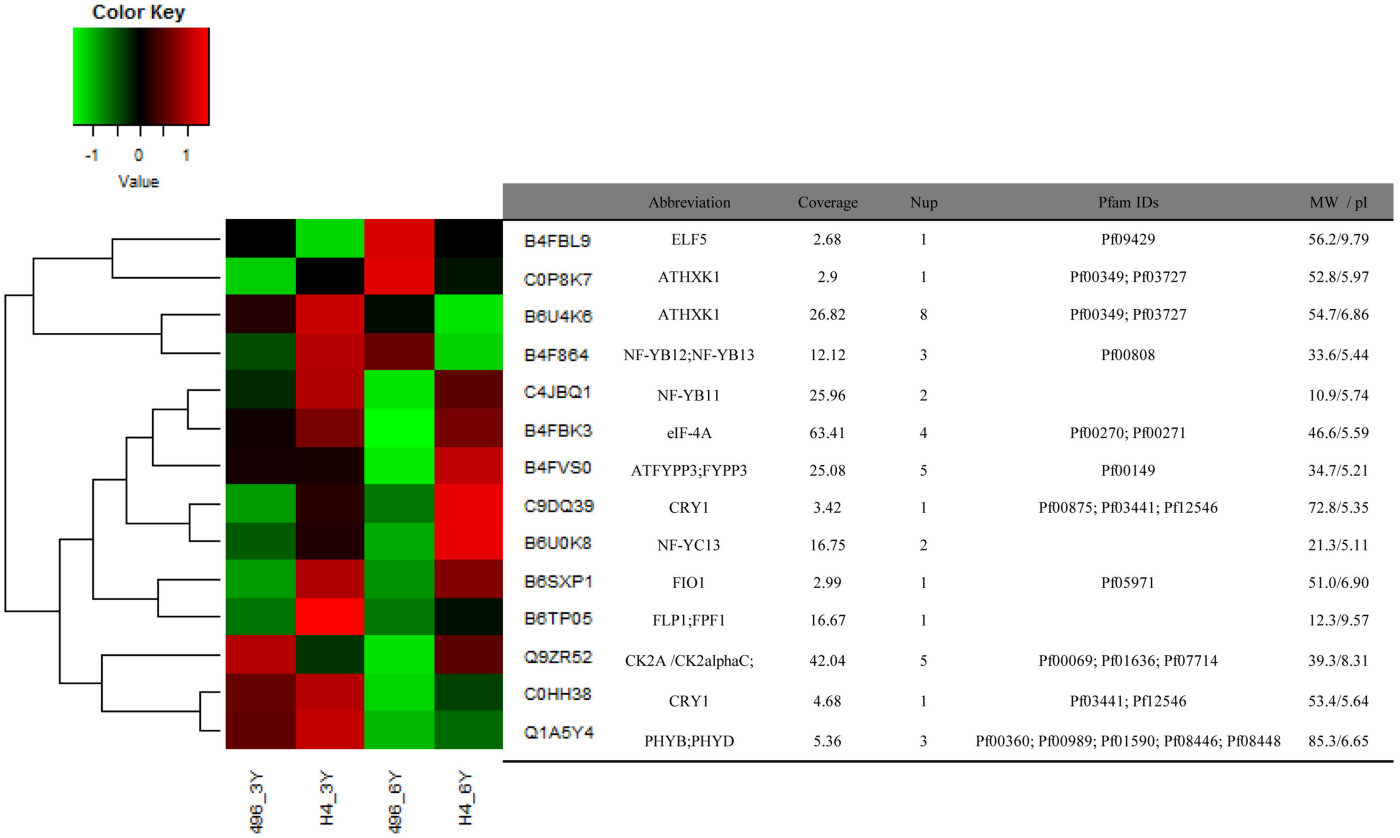
**Expression patterns of 14 identified proteins associated with light and photoperiod response in maize Huangzao4 (HZ4) and H496 grown under long-photoperiod conditions**. The heatmap was plotted using log2 values and scaled from a change of -1 to 1 log2 change.

**Figure 5:**
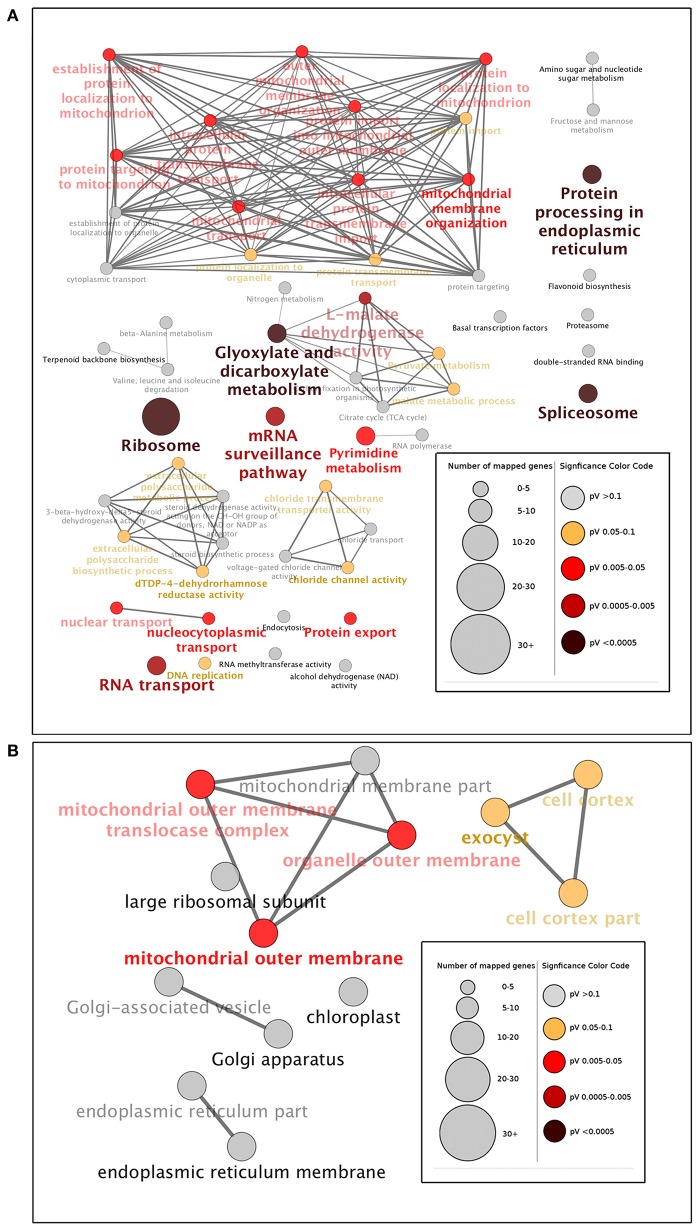
**Gene Ontology (GO) enrichment analysis of identified proteins at the three- and six-leaf stages of maize Huangzao4 (HZ4) and H496 grown under long-photoperiod conditions**. Different expressions of the protein interactome at the three- and six-leaf stages of HZ4 and H496 was analyzed using the Cytoscape plug-in ClueGo + Cluepedia to identify statistically enriched GO categories compared with the ClueGO maize reference genome. **(A)** GO categories searched include biological processes, molecular function, KEGG pathways, and **(B)** cell component. Nodes represent a specific GO term and are grouped based on the similarity of their associated proteins. Each node represents a single GO term and is color-coded based on enrichment significance (pV = *p*-value). Node size indicates the number of proteins mapped to each term. Edge thickness represents the calculated kappa score based on the number of proteins shared between terms. Functional groups are labeled by the most significant term in the group. Arrow indicates positive regulation.

Based on molecular functions, biological processes and KEGG pathways (Kanehisa and Goto, 2000), we also generated a GO annotation and KEGG network (KEGG-GO; Reference Genome Group of the Gene Ontology 2009) using the Cytoscape plug-in Cluego + Cluepedia (Bindea et al., 2009, 2013). Twenty terms were connected by 38 edges with the kappa scores, and showed considerable enrichment (*p* < 0.05) in the identified protein interactome (Figure 4). The most significant terms comprised those associated with protein processing in endoplasmic reticulum, splicesome, ribosome, glyoxylate, dicarboxylate metabolism, L-malate dehydrogenase activity, and RNA transport (Figure 5A). With regard to subcellular location, all proteins showed significant enrichment in the mitochondrial outer membrane (Figure 5B).

#### Protein synthesis

Protein turnover, which represents the balance between protein synthesis and degradation, is one of the many forms of regulation that is employed to achieve a unified cellular response (Reinbothe et al., 2010). In the present study, the most significant function enrichment pathway terms were ribosome, protein processing in endoplasmic reticulum, and RNA transport, which are involved in protein synthesis (Figure 5A). Missra et al. (2015) calculated the rate of protein synthesis by multiplying transcript abundance by translation state in *Arabidopsis* to show that high translation rates of TOC1 and LUX mRNAs at night may allow many related proteins to continue to repress transcription of the morning genes CCA1 and LHY, and of day genes such as GI and PRR9, which argued that it is plausible that differences in the waveform of protein synthesis rates may help to fine-tune circadian gene function (Missra et al., 2015). Recently, it has been reported that DNA replication during the cell cycle causes protein synthesis rates to show sharp, periodic jumps that can entrain the circadian clock in the cyanobacterium Synechococcus elongates (Paijmans et al., 2016). The present results provide additional evidence that protein synthesis has an important role in circadian regulations.

#### Metabolism process

As Figure 3 shown, Metabolism process constituted a high percentage (>70%) of the GO terms and Glyoxylate and dicarboxylate metabolism also elucidated significant enrichment in the KEGG-GO network (Figures 5A,B). These results indicated that amino acid metabolism may show distinct differences between HZ4 and H496 in response to LP. Connections between circadian clocks and carbon metabolism has been reported previously by Müller et al. (2014), and recently quantitative circadian phosphoproteomic analysis of *Arabidopsis* has revealed extensive clock control of key components in physiological, metabolic and signaling pathways and these findings showed new interaction networks that confer previously uncharacterized rhythms onto metabolism and physiology (Choudhary et al., 2015).

#### Post-transcriptional regulation

In 2011, Staiger D. and Köste T. have reviewed that post-transcriptional control in the circadian system of modern organisms, Drosophila, mammals, Neurospora, Chlamydomonas and *Arabidopsis* (Staiger and Köster, 2011). Notably, the next year alternative splicing (AS), as one type of post-transcriptional regulation, was reported as a way of linking the circadian clock to temperature response in *Arabidopsis* by AS of circadian gene CCA1(Park et al., 2012). Recently, Papasaikas and collaborators observed that GEMIN2, the only component of the SMN complex that is conserved from yeast to humans, controls the pace of the circadian clock under standard growth conditions in *Arabidopsis* by controlling the AS of TOC1 and other core clock genes (Papasaikas et al., 2015).

#### Mitochondrial outer membrane

In a study of the mitochondrial outer membrane in *Arabidopsis*, Duncan et al. (2011) developed a statistically rigorous quantitative proteomic workflow to confidently determine components of the outer mitochondrial membrane proteome of *Arabidopsis*. The proteins identified range from plant-specific proteins with unknown functions to proteins that have putative functions in mitochondrial signaling, morphology, and defense responses. In the present study, the mitochondrial outer membrane, as the only significantly enriched subcellular location, may be an important and novel location for proteins associated with photoperiod response (Figure 5B).

### Protein species expressed specifically related to photosynthesis in three-leaf stage or six-leaf stage in response to LP between HZ4 and H496

Under LP condition, some differentially expressed proteins identified in H496 compared with HZ4 exhibited similar proteome patterns at both the three- and six-leaf developmental stages (Figure 2A)., Such proteins included the Gibberellin receptor GID1L2 (B6TKC8), pollen-specific protein (B6TIS3), cryptochrome 2 (C9DQ39), and cyptochrome P450 super family protein (B4FQH2) (Table S 3). The majority of proteins identified in the two inbred lines differed between the three-leaf and six-leaf stages (Figure 2A, Tables S 4, S 5). In addition, photoperiod altered the expression of all differentially expressed proteins involved in carbon and energy metabolism, including ribosome, L-malate dehydrogenase activity, glyoxylate and dicarboxylate metabolism (Figure 5A). Some specially expressed proteins play a specific role in photosynthesis at both the three- and six-leaf stages (Figure 2A, Tables 2, 3). The three main classes were carbohydrates, amino acids, and lipids (Tables 2, 3), the protein amounts of these three metabolites (34/56, 60.7%) were higher in H496 compared with HZ4 at the three-leaf stage; but at the six-leaf stage only half of these proteins showed elevated expression. This result indicated that photosynthesis at the two developmental stages of HZ4 and H496 may show different regulatory mechanism responses to the LP condition.

**Table 2:** **Functional classification of identified proteins significantly differentially expressed at the three-leaf stage of maize HZ4 and H496 plants exposed to long-photoperiod conditions**.

**Accession No**.	**Protein species**	**MW/Pi**	**Nup**.	**Ratio**	***p*-value**	
**CARBOHYDRATE**						
B7ZWY9	Citrate synthase	62.7/7.18	1	1.313	0.02136	up
P80607	Alpha-1,4-glucan-protein synthase	177.7/6	3	1.414	0.00341	up
C0P5G0	Beta-amylase	49.3/6.95	1	0.632	0.00103	down
C0P7X9	Beta-galactosidase	33.1/8.12	2	0.284	2.06E-19	down
Q6VWJ0	Caffeoyl-CoA 3-O-methyltransferase 1	16.4/4.55	3	1.397	0.00474	up
O64909	Glucose-6-phosphate/phosphate translocator 2	46.8/9.74	1	0.745	0.03564	down
B6SRN7	Glucose-6-phosphate/phosphate translocator 2	23.7/8.48	1	0.69	0.00817	down
B4FZU8	Malate dehydrogenase	32.8/9.11	3	1.452	0.00162	up
B4FG53	Malate dehydrogenase	31.9/9.44	1	1.625	4.07E-05	up
B4FRJ1	Malate dehydrogenase	37.4/6.13	1	1.287	0.03272	up
F6MFD6	Malate dehydrogenase	54.4/8.87	3	1.307	0.02364	up
B6TTB7	Phosphoglycerate mutase family protein	13.2/6.13	1	0.736	0.02835	down
B6TYX7	Polygalacturonase inhibitor 1	94.4/6.23	1	1.304	0.02458	up
B6SS49	Protein kinase	15.4/9.25	1	1.363	0.00887	up
K7TPR9	Pyruvate kinase	21.9/9.45	1	1.328	0.01649	up
B4FCK7	Serine/threonine-protein phosphatase	32.3/8.48	2	0.712	0.01533	down
B6TKB3	Triose phosphate/phosphate translocator, non-green plastid,chloroplast	58.5/8.6	1	0.677	0.00541	down
**AMINO ACID**						
P25459	30S ribosomal protein S18, chloroplastic	51.3/5.36	3	1.279	0.03714	up
B4G1F2	39S ribosomal protein L12	47.6/5.73	1	1.276	0.03919	up
B4FID1	40S ribosomal protein S17-4	18.5/4.2	1	0.735	0.0283	down
B4FG22	40S ribosomal protein S25-1	42.8/9.7	2	0.65	0.0021	down
B4FZW6	50S ribosomal protein L24	29.8/9.14	1	1.348	0.01162	up
O24415	60S acidic ribosomal protein P2B	18.1/4.75	2	1.264	0.04737	up
B4FPT8	60S ribosomal protein L10-3	51.6/6.76	1	0.736	0.02904	down
B6SX76	60S ribosomal protein L18a	16.2/9.77	2	0.727	0.02311	down
B6TPG2	60S ribosomal protein L26-1	40/9.66	1	0.728	0.02353	down
B4FTA3	60S ribosomal protein L27	22.9/5.2	1	0.72	0.01916	down
B6TMH0	60S ribosomal protein L27	25.5/8.22	4	1.277	0.03855	up
B4FQD7	60S ribosomal protein L27a-2	14.1/8.65	1	1.304	0.02485	up
B6T4H7	60S ribosomal protein L27a-3	78.5/6.23	1	1.286	0.03319	up
B4FXX2	60S ribosomal protein L34	34.5/5.78	1	1.269	0.04369	up
B4FHI7	60S ribosomal protein L38	8.7/12	1	0.733	0.0266	down
B6TNB0	60S ribosomal protein L6	13.2/4.54	1	1.297	0.0276	up
B4FSG3	60S ribosomal protein L7-1	6.3/9.16	1	0.734	0.0271	down
K7TWD9	Chlorophyll a-b binding protein 4	42.2/9.79	3	1.423	0.00287	up
B4FV94	Chlorophyll a-b binding protein 4	39.6/5.9	2	1.33	0.01582	up
Q00827	Chlorophyll a-b binding protein 48, chloroplastic	52.7/8.47	1	1.502	0.00058	up
B7ZXB7	Coatomer subunit gamma	86.2/6.93	1	1.321	0.0186	up
B4G147	DNA-directed RNA polymerase II 19 kDa polypeptide	28.1/6.13	2	0.688	0.00757	down
K7USA4	DNA-directed RNA polymerase	94.8/6.04	1	1.264	0.04723	up
B6SHX9	Histone H2A	18.6/9.85	2	1.414	0.00339	up
B6TFY8	Histone H4	27.1/8.48	2	6.586	6.12E-57	up
**LIPIDS AND FATTY ACIDS**						
B6SXI7	Phosphatidylinositol-4-phosphate 5-kinase 9	21.6/7.9	1	0.713	0.0158	down
P60138	Photosystem II reaction center protein L	79.8/5.69	2	1.281	0.03625	up
B6STM5	Omega-3 fatty acid desaturase	64.8/6.16	1	1.365	0.00849	up
B6TN48	3-hydroxy isobutyryl-CoA hydrolase/catalytic	31.3/4.6	1	1.307	0.02366	up
B6T8T5	3-oxoacyl-reductase	57.5/8.25	1	0.661	0.00305	down
B4FFE7	Acyl carrier protein	8/12.02	1	1.376	0.0069	up
B6U6S2	Acyl-CoA synthetase long-chain family member 3	8/4.59	3	1.471	0.00111	up
B6UEF8	ATP binding protein	37.9/8.27	2	0.757	0.04689	down
K7VCM2	Putative RING zinc finger domain superfamily protein	7.2/4.35	1	1.532	0.00031	up
K7TPR9	Pyruvate kinase	21.9/9.45	1	1.328	0.01649	up
O82579	Ribosomal protein L26 (Fragment)	71.6/5.6	2	0.682	0.00633	down
**PHOTOPERIOD**						
Q6VWJ0	Caffeoyl-CoA 3-O-methyltransferase 1	16.4/4.55	3	1.397	0.00474	up
Q8LK10	DNA methyltransferase DMT106	18.1/8.94	1	0.125	6.13E-50	down
O82579	Ribosomal protein L26 (Fragment)	71.6/5.6	2	0.682	0.00633	down

Annotations were obtained from the UniProtKB/Swiss-Prot databases (http://www.expasy.org/).

**Table 3:** **Functional classification of identified proteins significantly differentially expressed at the six-leaf stage in maize HZ4 and H496 plants exposed to long-photoperiod conditions**.

**Accession No**.	**Protein species**	**MW/pI**	**Nup**.	**Ratio**	***p*-Value**	
**CARBOHYDRATE**						
Q9SBJ3	6-phosphogluconate dehydrogenase isoenzyme A	19/5.07	1	1.32	0.0466823	up
C0PIW1	Glucose-6-phosphate 1-dehydrogenase	68.3/8.82	3	1.623	0.000531937	up
B4FAK9	Glyceraldehyde-3-phosphate dehydrogenase, cytosolic	43.3/8.27	3	1.317	0.048407	up
B6TZ09	Limonoid UDP-glucosyltransferase	51.9/4.88	1	1.781	3.64E-05	up
P49081	Malate synthase, glyoxysomal	61.6/6.64	1	0.594	0.00275703	down
K7UJM1	Putative O-Glycosyl hydrolase superfamily protein	32.9/5.74	1	0.637	0.00973655	down
B6TV55	Stem 28 kDa glycoprotein	32.1/9.13	1	0.397	1.13E-07	down
B6SMV7	Triosephosphate isomerase	25/7.9	1	0.527	0.00023741	down
B6TY47	UDP-glucuronic acid decarboxylase 1	45.3/9.41	1	0.595	0.0029176	down
**AMINO ACIDS**						
B4FQ29	26S proteasome non-ATPase regulatory subunit 13	43.8/5.69	3	0.665	0.0192674	down
P06586	30S ribosomal protein S3, chloroplastic	25.9/9.74	8	1.386	0.0195563	up
B6T1J3	40S ribosomal protein S13	16.9/10.45	1	1.326	0.0433027	up
B6TJ93	40S ribosomal protein S15a	14.7/9.82	2	1.356	0.0290148	up
B6TM74	50S ribosomal protein L27	19.6/10.36	2	1.329	0.0417262	up
K7UI47	50S ribosomal protein L35	8/11.4	2	1.316	0.0489937	up
P46252	60S acidic ribosomal protein P2A	11.4/4.28	1	0.621	0.00624524	down
B6SPH4	60S ribosomal protein L29	6.9/10.68	1	1.502	0.00360954	up
B6T098	60S ribosomal protein L34	13.7/11.49	1	0.633	0.0087787	down
B6SX73	60S ribosomal protein L35	14.3/11.28	3	1.395	0.0172634	up
B6SIY6	60S ribosomal protein L44	12.1/10.2	3	1.324	0.0443997	up
C0PI96	DNA-directed RNA polymerase	40.6/9.09	2	0.54	0.000402232	down
B4G237	Histone H2A	14/10.05	2	0.403	1.82E-07	down
B4FYZ0	Histone H2B	16.1/10.05	1	1.434	0.00987423	up
B6T1G5	Histone H2B	16/10.08	1	1.352	0.0308925	up
P49120	Histone H2B.4	15.2/10.02	1	0.669	0.0210391	down
**LIPIDS AND FATTY ACIDS**						
B4FJG4	DNA-directed RNA polymerase II 8.2 kDa polypeptide	8.3/5.06	1	0.556	0.000755199	down
B6U6C1	Flavonol synthase/flavanone 3-hydroxylase	44.8/5.02	1	0.487	3.70E-05	down
C0PIW1	Glucose-6-phosphate 1-dehydrogenase	68.3/8.82	3	1.623	0.000531937	up
B4FAK9	Glyceraldehyde-3-phosphate dehydrogenase, cytosolic	43.3/8.27	3	1.317	0.048407	up
B7ZZX9	Lipoxygenase	98.1/6.71	6	1.761	5.15E-05	up
B6TCR8	Protein binding protein	34.6/4.13	3	0.532	0.000287393	down
B6U0J9	Protein binding protein	71.9/4.97	3	0.632	0.00835835	down
B4FWN6	Putative RING zinc finger domain superfamily protein	36.1/7.77	1	0.668	0.0205105	down
**PHOTOPERIOD**						
P06586	30S ribosomal protein S3, chloroplastic	25.9/9.74	8	1.386	0.0195563	up
K7URS7	Putative cytochrome P450 superfamily protein	64.2/7.97	4	0.68	0.0271578	down

Annotations were obtained from the UniProtKB/Swiss-Prot databases (http://www.expasy.org/).

#### Carbohydrate

The proteins involved in carbon assimilation showed significant changes in abundance under LP. A previous report indicated that enzymes functioning during the reduction phased of the Calvin-Benson cycle accumulated to higher levels shoot tips under LP, whereas the level of enzymes involved in carboxylation and regeneration phases was increased in shoot tips under SD condition (Victor et al., 2010). In the present study, triose phosphate isomerase (B6AMV7), an important enzyme in the Calvin-Benson cycle, was less abundant in LP leaves (Table 3). This result contradicts previous observations and may be owing to differences between tissues, or the mechanisms of the response to LP in leaf may be more complex and involve additional regulators than compared with that in the shoot tips.

Potentially increased availability of carbohydrate under LP may be the reason for elevated accumulation of enzymes responsible for glycolysis, such as malate dehydrogenase (B4FZU8, B4FG53, B4FRJ1, F6MFD6; Table 3), which are involved in the pathway following glycolysis and were also more abundant in leaves. In *Arabidopsis*, the activities of enzymes involved in the glycolysis pathway were decreased in response to a shortened photoperiod, whereas activity of enzymes participating in photosynthesis and starch synthesis remained high.

As a diurnally regulated carbohydrate, sucrose content increases during light conditions and decreases during dark conditions, consistent with previously reports for other plants, such as potato (Urbanczyk-Wochniak et al., 2005). Glucose-6-phosphate, which is responsible for sucrose biosynthesis as well as degradation, exhibited a similar pattern to that of sucrose (Urbanczyk-Wochniak et al., 2005). Hoffman et al. (2010) reported that diurnal fluctuations were regulated by several Krebs-cycle intermediates in pool sizes. In our study, we measured the sucrose and glucose contents in the leaf of HZ4 and H496 at the three- and six- leaf stages. The sucrose and glucose contents in H496 were slightly lower than those of HZ4 at the three-leaf stage, but higher at the six-leaf stage (Figures S 1A, B). This finding indicated that sucrose and glucose showed homeostatic changes in response to LP in the development of the two lines. Malate dehydrogenase showed an activated pattern at the three-leaf stage in H496, with an increased level compared to HZ4. Significant differences also observed for several proteins involved the metabolites between the two species at both stages (Tables 2, 3). These results provide new evidence to further verify carbohydrate will mediated the circadian response.

#### Chloroplast proteins

Adequate light harvesting for photosynthesis is closely related with the abundance of chloroplast proteins, such as chlorophyll a/b binding protein, which is responsible for energy transfer in the reactive center in photosystem II (Kovács et al., 2006). This protein is responsible for balancing the distribution of excitation energy between photosystems: I and II (Kovács et al., 2006). Interestingly, one of these genes encoding chloroplast a/b binding protein was homologous to known genes that are responsible for the floral transition or morphology and the circadian rhythm photoperiod response in *maize, rice* and *Arabidopsis* (Coles et al., 2010). In the present study, expression of two chlorophyll *a*/*b* binding proteins, K7TWD9 and B4FV94, was increased in H496. Thus, these two proteins are predicted to be involved in the circadian rhythm response, but confirmation requires further investigation.

#### Ribosomal proteins

We detected 27 ribosomal proteins, of which 16 proteins were upregulated and 11 proteins were downregulated (Tables 2, 3). Previously, the ribosomal protein gene L34 (*rpL34*), which encodes a cytoplasmic ribosomal protein with high homology to the rat 60S r-protein, was isolated from a genomic library of tobacco (*Nicotiana tabacum* cv. Xanthi-nc), and histochemical GUS staining showed that rpL34 promoter activity was high in actively growing tissues, including various meristems, floral organs and developing fruits (Dai et al., 1996). In the present study, the ribosomal protein L34 was downregulated in HZ4 only at the six-leaf stage, which is the stage at which shoot apex morphology changes (Wu et al., 2008). Thus, the early flowering habit of HZ4 may be caused by activation of the ribosomal protein. Conversely, translation, especially the production of ribosomal proteins, is positively correlated with the abundance of phosphorylated S6 protein (Williams et al., 2003; Turck et al., 2004). The phosphorylation of L29-1, a 60S ribosomal protein, is enhanced under moderate “day time,” and the possibility of diurnal regulation of translation in plants is indicated by differential phosphorylation of at least three ribosomal proteins: the 40S ribosomal proteins S6-1 and S6-2, and the 60S ribosomal protein L29-1 (Turkina et al., 2011).

In the current study, a higher number of ribosomal proteins were upregulated in H496, and the 60S ribosomal protein L29 showed a higher expression level in H496, which is consistent with the above-mentioned report (Turkina et al., 2011). However, information on the exact mechanisms to explain increased protein production in the light phase of the photoperiod is extremely limited. The present findings shed some light on this conundrum by indicating that a portion of the enhanced protein synthesis may result from diurnal regulation of translation by complex combinatorial phosphorylation of ribosomal proteins. Overall, whether the ribosomal proteins are upregulated or downregulated, it would be an important cue in the regulation of flowering and photoperiod response, but the molecular function and regulatory mechanism for each ribosomal protein are poorly known and require further investigation in the future.

### Expression pattern of iron metabolism-related proteins under LP condition

Anti-oxidative molecules, such as ferritin, are essential to detoxify reactive oxygen species or buffer irons to prevent oxidative stress (Ravet et al., 2009). Iron is a critical component for the function of many photosynthetic proteins, and iron deficiency causes an extended free-running period of rhythm changes and increases the production of reactive oxygen species (Salome et al., 2013). Thus, a higher level of ferritin 1 may correspond to enhanced detoxifying process of reactive oxygen species and distinct reactions to LP treatments. In the present research, the increase in accumulation of ferritin 1 (K7U2L3) of inbred line H496 leaves at six-leaf stage was higher than that in HZ4 under LP (Figure S1 C), suggesting that the strength of photosynthesis and the production of photosynthetic protein were higher in H496 compared with HZ4.

### Expression pattern of circadian-associated proteins under LP condition

Sorts of categories of circadian-associated proteins have been identified, and the expression patterns of 14 circadian associated proteins were examined (Figure 4). We found that eight proteins were upregulated in HZ4 in three-leaf stage, and five in six-leaf stage. But compared with that in H496, many proteins were elevated in HZ4 of three-leaf (11/14, 78.57%) and six-leaf stage (9/14, 64.28%), except that Q9ZR52 (CK2 alpha) were downregulated at the three-leaf stage, as well as C0P8K7 (AtHXK1), B6U4K6 (AtHXK1) and B4F864 (NF-YB12; NF-YB13) in the six-leaf stage. Interestingly, the amount of B4FBL9 (*ELF5*) decreased at both stages while B4FVS0 (ATFYPP3) and Q1A5Y4 (PHYB; PHYD) have no change separately in three-leaf and six-leaf stage.

**Figure 6:**
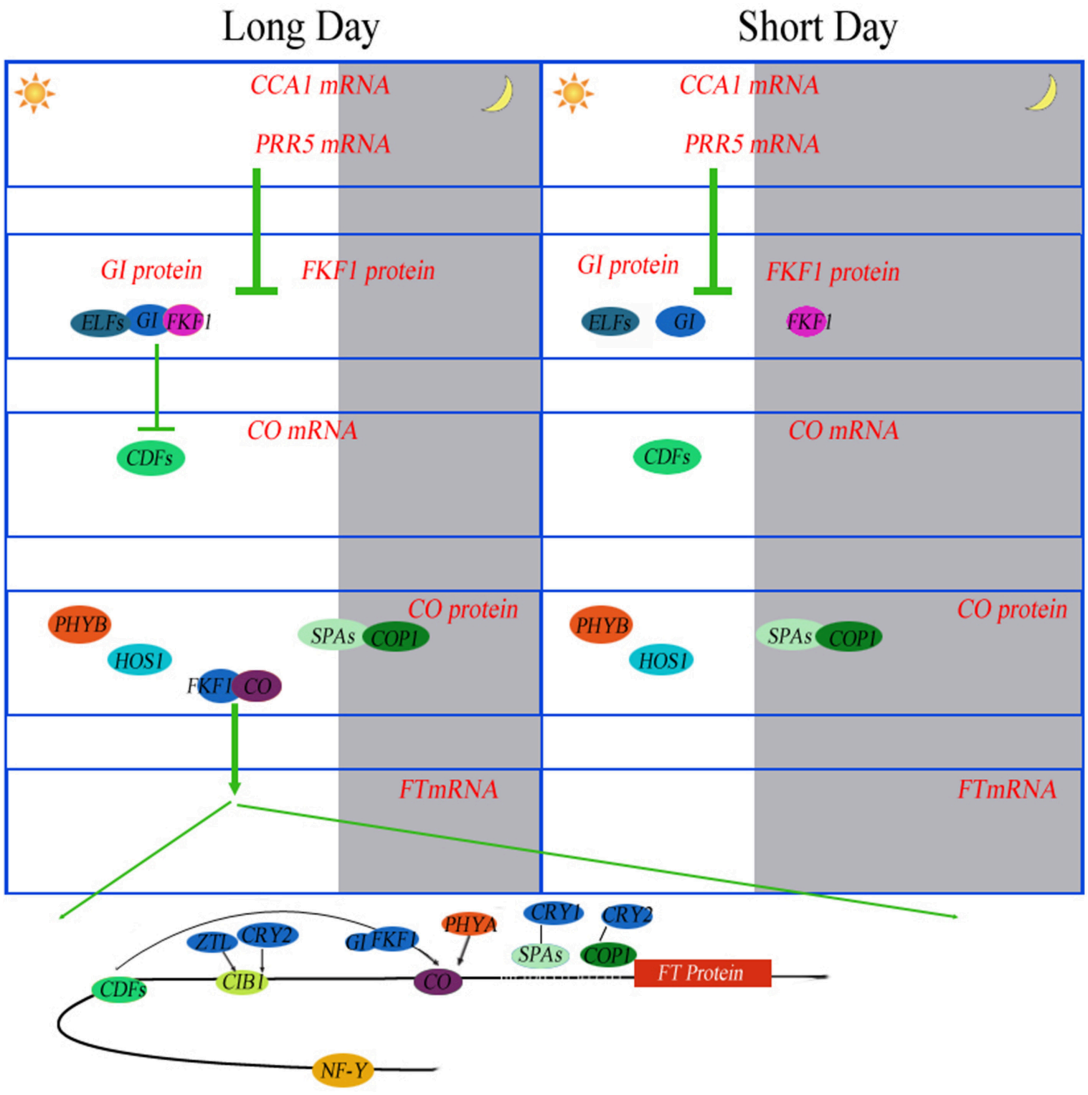
**Photoperiodic regulation of FT induction in ***Arabidopsis*****. Under long days (LD), the abundance of *CCA1* transcripts oscillates throughout the day. CCA1 and its homolog LHY bind to the promoters of *PRR5, FKF1* and *GI* to repress their expression in the morning. PRR5 also negatively controls the expression of *CDF* genes. Daily expression profiles of CDF1 are regulated by the FKF1–GI complex. The same mechanism of degradation of CDFs by the FKF1–GI complex is also exerted on the FT promoter. FKF1 physically interacts with CO protein to stabilize it. PHYA also stabilizes CO protein. Stabilized CO protein binds to the FT promoter to activate FT expression. NF-Y complex enhances the binding of CO protein to the FT promoter. CIB1 is activated by blue light absorbed by CRY2 and stabilized by blue light absorbed by ZTL. CIB1 directly activates the expression of FT in the afternoon. These proteins prevent flowering under unfavorable conditions, such as short days (SD). Under SD, the expression peaks of FKF1 and GI do not coincide. In the absence of the FKF–GI complex, CO expression is continuously suppressed by CDF proteins during the day.

Post-translational regulation of CONSTANS (CO) protein is another key element of the photoperiodic induction of *FLOWERING LOCUS T* (*FT*) transcription (Möglich et al., 2010). Phytochrome is an important regulator coordinating downstream signaling components, and many studies have focused on elucidating novel components involved in light signal transduction (Paul and Khurana, 2008). The combination of CO with NF-Y transcription factors activates FT during floral initiation, which is dependent on photoperiod (Kumimoto et al., 2010). An *ELF5* (B4FBL9) mutation, *elf5*, partially suppresses the photoperiod pathway and causes early flowering under SD, suggesting that *ELF5* controls flowering independent of *FLOWERING LOCUS C* (*FLC*), a floral repressor upon which many of the flowering pathways converge (Noh et al., 2004). Moreover, ELF4 regulates the access of GIGANTEA (GI) to chromatin by sequestering GI from the nucleoplasm into subnuclear bodies preferentially during the night, thus restricting its ability to bind to the *CO* promoter (Kim et al., 2013). Recently, the FPF1 (B6TP05) class genes have been explored, which may act as a regulator of flowering and the formation of wood in poplar (Hoenicka et al., 2012). To attain synchrony with day and night, the clock is entrained via the red/far-red-absorbing PHYTOCHROMES (PHYA-PHYE), the blue light-absorbing CRYPTOCHROMES (CRY1 and CRY2), and the LOV (LIGHT, OXYGEN, VOLTAGE) domain proteins ZEITLUPE (ZTL), FLAVIN BINDING, KELCH REPEAT, F-BOX1 (FKF1), and LOV KELCH PROTEIN 2 (LKP2) (Devlin, 2002; Fankhauser and Staiger, 2002) (Figure 6).

Some additional proteins related to plant circadian rhythms have been identified. overexpression of *Arabidopsis* hexokinases (AtHXK1, C0P8K7, and B6U4K6) in tomato plants may reduce photosynthesis, inhibit growth and accelerate senescence (Dai et al., 1999), These results indicate that the activity of endogenous hexokinase is not a factor limiting growth rate, but functions to regulate photosynthesis in photosynthetic tissues. Overexpression of AtHXK1 in tomato plants also reduced the chlorophyll content. From this result, we assume that HXK, as a sugar phosphorylation enzyme, is a negative regulator of photosynthesis. The study by Miao et al. (2013) reinforces and extends the argument that the promoted biosynthesis of aliphatic glucosinolate by glucose is involved in HXK1- and/or RGS1-mediated signaling through the transcription factors MYB29, MYB28 and ABI5. In a previous study we demonstrated that in transgenic *Arabidopsis* plants, ZmHd6, encoding a protein similar to the *Arabidopsis* of CASEIN KINASE2 alpha subunit (CK2 alpha, Q9ZR52), affected the flowering time through the photoperiodic pathway in maize (Ku et al., 2011). PSEUDO-RESPONSE REGULATOR 7 (PRR7), which is considered a “morning-expressed” gene, was isolated recently (Haydon et al., 2013). By inhibiting photosynthesis, the authors described that endogenous fluctuations in sugar levels supplied feedbacks at metabolic level to circadian oscillator via PRR7. In addition, *ppr7* mutants are insensitive to the oscillations of sucrose levels during circadian rhythms. Consequently, in *Arabidopsis*, robust circadian rhythms are stringently maintained by photosynthesis, demonstrating that the circadian clock is regulated by metabolism to a large extent (Haydon et al., 2013).

## Conclusions

In this study, 5259 proteins were detected in maize leaves in the inbred lines HZ4 and H496. On the basis of MS/MS identification, 943 proteins were expressed differentially between HZ4 and H496, and those proteins were commonly shared by the newly expanded leaves from three- and six-leaf stages. Fourteen circadian associated proteins were also examined. The protein expression patterns of the inbred lines differed significantly even though the two lines share a similar genetic background. The proteomic changes in the maize leaf induced by LP treatment were highly function-specific, such as endoplasmic reticulum, splicesome, ribosome, glyoxylate, dicarboxylate metabolism, L-malate dehydrogenase activity, and RNA transport. The protein species differentially expressed between HZ4 and H496 were associated with photosynthesis including carbohydrate, chloroplast and ribosomal proteins at the three- or six-leaf stages in response to LP. To adapt to the outside environment, the phase of rhythms are adjusted in response to environmental signals, such light and external sugar supplement. The regulation patterns of light and circadian-associated protein under LP condition are discussed. The iron metabolism-related proteins and circadian-associated protein, such as K7U2L3, C0P8K7, and Q9ZR52, may mediate the photoperiodic pathway. The results offer novel insights into the influence of LP and provide additional information on the mechanism of circadian response in short-day plants at the proteomic level.

## Author contributions

YC, LW, and LT conceived and designed the experiments. LW, LT, JZ, SW, PL, HZ, and HL Performed the experiments. LW and JZ Analyzed the data. YC and SW contributed reagents, materials and analysis tools. LW, YC, and LT wrote the manuscript.

### Conflict of interest statement

The authors declare that the research was conducted in the absence of any commercial or financial relationships that could be construed as a potential conflict of interest.

## Supplementary material

Figure S 1 **Accumulation content of sucrose (A), glucose(B) and Fe(C) between HZ4 and H496 in three- and six-leaf stages**. Two-Way ANOVA followed by Student-Neuman-Keuls *post-hoc* test (***p* < 0.01,**p* < 0.05).

Table S 1 **All proteins were identified by MS/MS in 3-leaf stage and 6-leaf stage of HZ4 and H496 under LP condition**.

Table S 2 **All peptides of identified proteins in three- and six-leaf stages of HZ4 and H496 under LP condition were identified by MS/MS**.

Table S 3 **Differentially expressed protein in H496 compared to HZ4 in both two stages**.

Table S 4 **Different expressed specially proteins in H496 compared to HZ4 in 3-leaf stage**.

Table S 5 **Different expressed specially protein in H496 compared to HZ4 in 6-leaf stage**.

Table S 6 **Two-way analysis of variance of proteins differentially expressed at the three- and six-leaf stages in HZ4 and H496 grown underLP conditions**. IL, inbred lines; DS, developmental stages.
