# Supplementary figures and images for: Comparative Proteomic Analysis of the Response of Maize (Zea mays L.) Leaves to Long Photoperiod Condition
Source: Front Plant Sci. 2016 Jun 2;7:752. doi: 10.3389/fpls.2016.00752 (PMC4889979; doi:10.3389/fpls.2016.00752)

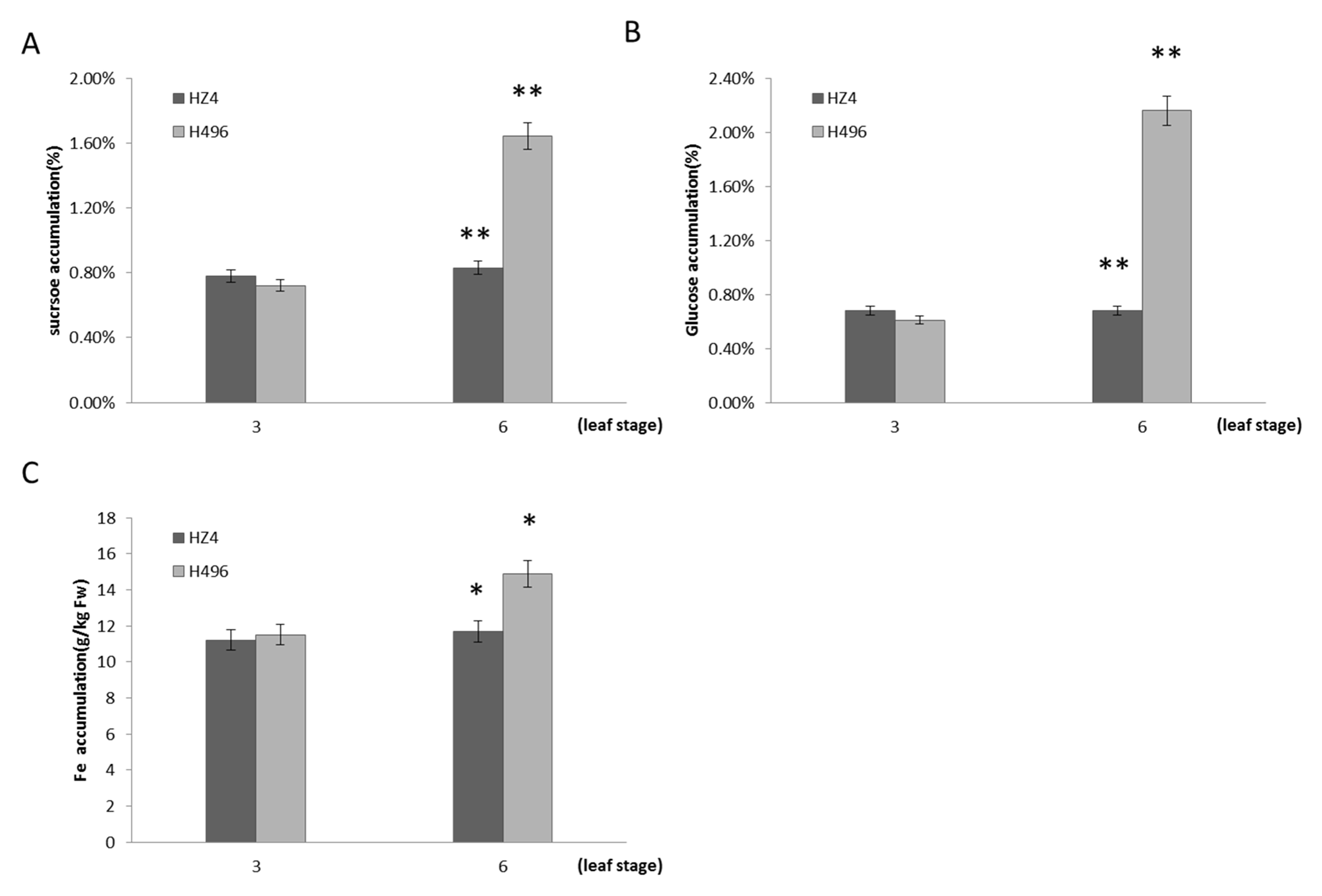

Supplement: Figure S 1 — Accumulation content of sucrose (A), glucose(B) and Fe(C) between HZ4 and H496 in three- and six-leaf stages. Two-Way ANOVA followed by Student-Neuman-Keuls post-hoc test (**p < 0.01,*p < 0.05). [file Image1.tif]
